# Notes on the Atlantic City Meeting

**Published:** 1904-08

**Authors:** 


### NOTES ON THE ATLANTIC CITY MEETING.

Judging by the programme for the section on Materia Medica, Pharmacy and Therapeutics at Atlantic City, there is a Senagambian in the wood-pile. Therefore the ethical wiseacres of the Association will have to be wide-awake, or this section will be used by the shrewd and slick advertisers of "Goo Goo" foreign unpronouncable synthetics to a surprising extent. One who is listed to read two papers, has in his own publication practically advocated substitution by druggists and is generally supposed to be interested commercially with the manufacturers whose wares he puffs so vigorously. The American Medical Association has a dignity to maintain and should not allow a blatant, ignorant pomposity to push itself into their councils and divert the valuable purposes of the section on materia medica into a medium for self-advertising, the display of chemical or pharmaceutical ignorance, questionable favoritism, or the splenetic vagaries of a self-constituted "Critic and Guide." Let there be a careful censorship of the articles read at this section, so that its purposes may not be diverted.

Speaking of splenetic vagaries. Chemists, as a profession, are not especially addicted to this trouble, but, when they delve into matters foreign to their science and begin to interfere with the doctor, or to establish standards not warranted by medical practice, then their vagaries are a nuisance. For instance, who cares whether a "synthetic" is compounded before or after the crystallization of its components. The word synthetic itself indicates that it is a compound. Dr. Foster, the editor of the *New York Medical Journal,* once said: "I don't care a Rappahannock whether a drug is a compound or a derivative, so long as it does the work." This we believe voices the real opinion of the medical profession at large.

The advent of pharmaceuticals made from coal-tar was heralded by antipyrin, a patented preparation which is said to have been immensely profitable. This was followed by phenacetin, another patented preparation which has been very profitable in America, as the proprietors have been able to charge American consumers nearly eight hundred per cent. above their price in other countries. For some inscrutable reason this proprietary remedy has had an enormous amount of free advertising in medical journals, generally to the detriment of American manufacturers, who are liberal supporters of the advertising pages.

There are chemical cranks who are "know-it-alls." They conclude that some manufacturer is gaining too much popularity, and disregarding the real virtue of his remedy, proceed to "analyze" it, with the deliberate purpose of betraying the confidence of the medical profession and incidentally gaining reputation as wise chemists. Woe betide the owner of a proprietary preparation, if one of these "chemical cranks" gets after him; he sticks at nothing; often being financially irresponsible and having access to the medical and pharmaceutical press, he will analyze(?) and damn the most valuable drug known to the medical profession. These "wise guys" most affect a foreign attitude. A drug "made in Germany," or anywhere except in their own country, is like Cæsar's wife, and being above suspicion of commercialism, its formula is immediately enshrouded with the hieroglyphics of chemical nomenclature so cunningly devised that the average well-read physician is lost in wonder that nature's alembic could produce an alchemic brain capable of evolving such a wonderful "synthetic" result.—*Medical Mirror, June, 1904.*

